# A Case Report of a Giant Renal Angiomyolipoma Complicated by Perinephric Hematoma in a Patient With Tuberous Sclerosis

**DOI:** 10.7759/cureus.88627

**Published:** 2025-07-23

**Authors:** Sanjana Janumpally, Alexander Konopnicki, Roopkiran Panesar, Bagi Jana

**Affiliations:** 1 Internal Medicine, University of Texas Medical Branch, Galveston, USA; 2 Hematology and Oncology, University of Texas MD Anderson Cancer Center, Galveston, USA

**Keywords:** everolimus, oncology, perinephric hematoma, renal angiomyolipoma (aml), tuberous sclerosis

## Abstract

Tuberous sclerosis (TSC) is an autosomal dominant neurocutaneous disorder where pathogenic variants cause overactivation of the rapamycin (mTOR) pathway, leading to tumor formation. These benign tumors, or hamartomas, occur in multiple organs, including the brain, skin, eyes, kidneys, lungs, and liver. Renal angiomyolipomas (AMLs) occur frequently in TSC and are highly vascular tumors primarily composed of blood vessels, smooth muscle, and mature adipose tissue. Renal AMLs greater than 10 cm are rarely seen and are considered “giant” AMLs. Everolimus, an mTOR inhibitor, is a treatment option to slow the growth of renal AMLs. Renal tumors, especially renal AMLs, are the most common cause of perinephric hematomas. For renal AMLs with a high risk of bleeding, embolization can be used to delay renal decline. We present the case of a young patient with a history of TSC complicated by bilateral renal AMLs that required right-sided nephrectomy and chronic kidney disease stage four who presented with progression of her known left-sided renal AML to giant status and perinephric hematoma following both everolimus treatment and multiple embolizations. The purpose of this case report is to reinforce the importance of routine oncology follow-up to prevent the progression of renal AMLs and to highlight the multidisciplinary approach of multiple specialties to preserve the quality of life and renal function of our young patient with a rarely reported “giant” AML.

## Introduction

Tuberous sclerosis (TSC) is a rare autosomal dominant neurocutaneous disorder that affects approximately one in 6,000 live births [1]. It is characterized by the development of benign tumors, or hamartomas, in multiple organs, including the brain, skin, eyes, kidneys, lungs, and liver [2]. TSC is caused by heterozygous pathogenic variants in either the TSC complex subunit 1 (TSC1) or the TSC complex subunit 2 (TSC2) tumor suppressor genes, which lead to overactivation of the rapamycin (mTOR) pathway and tumor formation in multiple organs [3]. Renal angiomyolipomas (AMLs) are highly vascular tumors primarily composed of blood vessels, smooth muscle, and mature adipose tissue [4]. Most AMLs are asymptomatic and do not require treatment. When symptomatic, features related to hemorrhage are most common and include abdominal or flank pain, hematuria, hypertension, anemia, and kidney function impairment [5]. For patients with AMLs growing rapidly (>2.5 mm per year) and preserved kidney function (eGFR > 60 mL/min/1.73 m^2^), recommended treatment is with an mTOR inhibitor such as everolimus [6]. However, intervention with selective artery embolization is appropriate for lesions that are at high risk for bleeding [7]. The incidence rate of a renal AML is around 0.3%-3% of the general population, but a "giant" renal AML is even more rare [8]. Renal AMLs greater than 10 cm are considered “giant” [7]. Giant AMLs in patients with advanced chronic kidney disease can pose significant treatment dilemmas. Herein, we present the case of a 33-year-old female with a history of TSC and previous right-sided nephrectomy due to giant renal AML presenting with left-sided giant renal AML with perinephric hematoma.

## Case presentation

**Figure 1:**
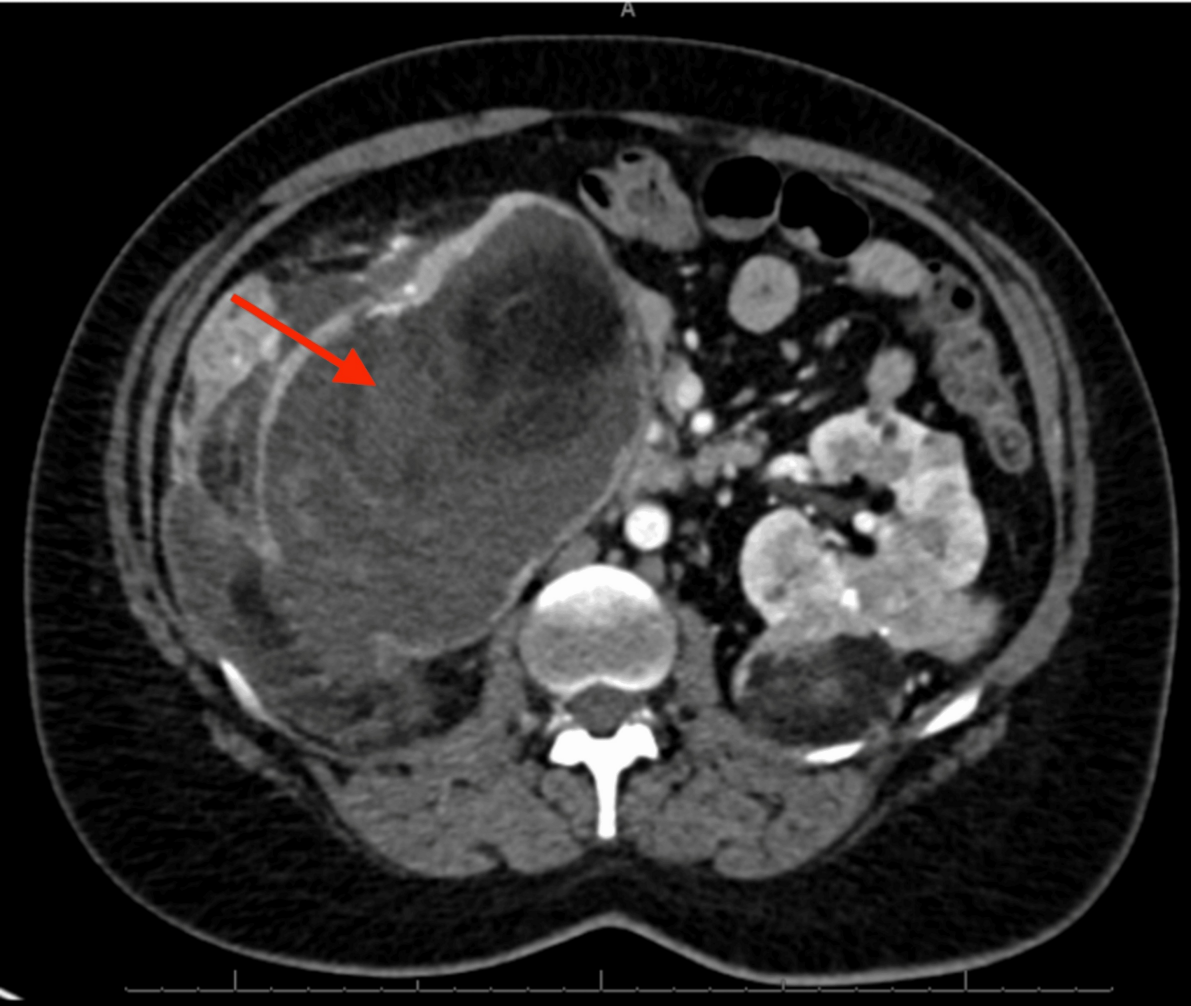
CT abdomen and pelvis: a right renal angiomyolipoma with no recognizable renal parenchyma measuring 16.1 x 12.0 x 19.6 cm with a right perinephric hematoma in the axial plane

**Figure 2:**
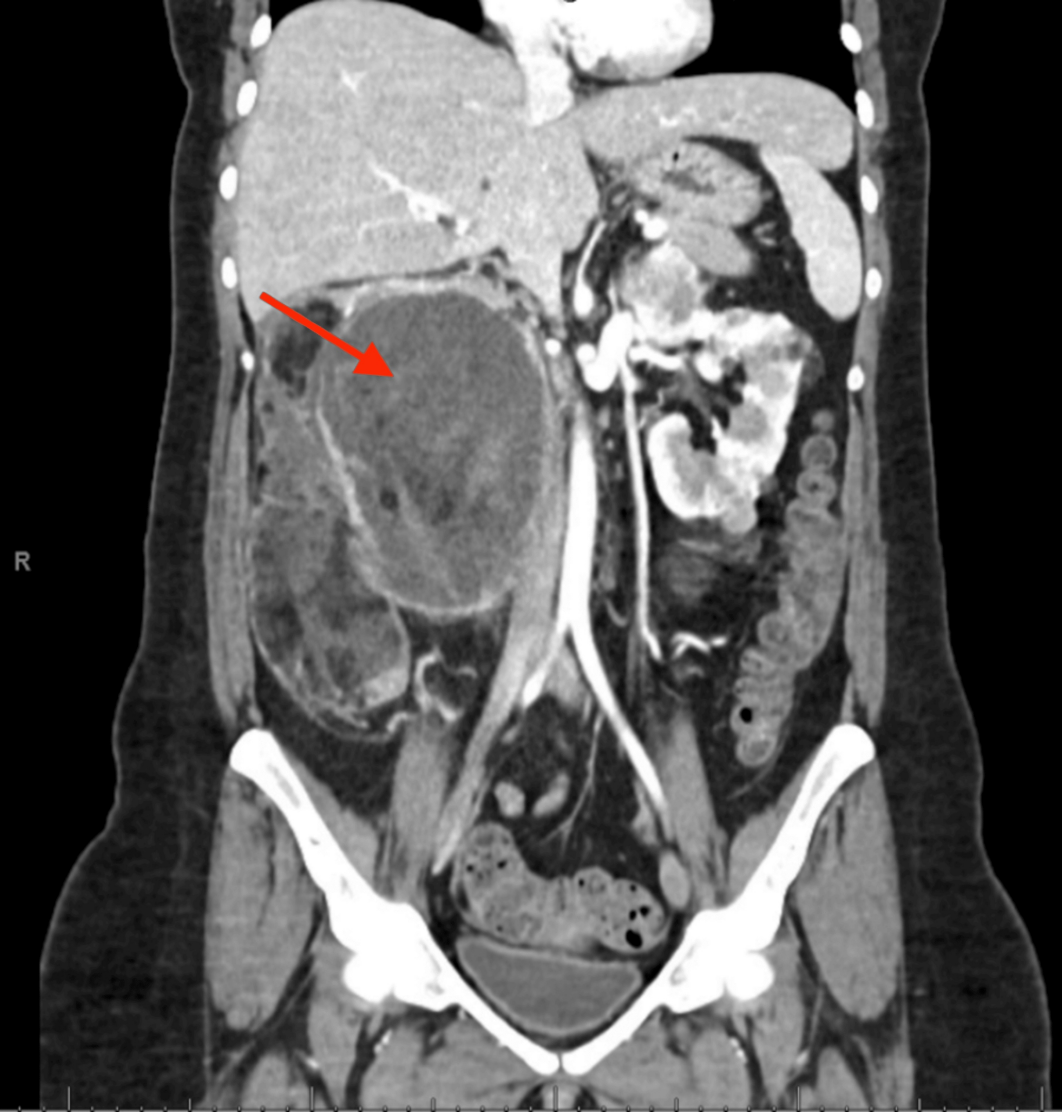
CT abdomen and pelvis: right renal angiomyolipoma measuring 16.1 x 12.0 x 19.6 cm in the coronal plane

A 33-year-old female with a past medical history of TSC, bilateral renal AMLs requiring right-sided nephrectomy four years prior, chronic kidney disease (CKD) stage 4, giant cell astrocytoma, and seizures was admitted for five days of gradually worsening left-sided abdominal pain and swelling. Associated symptoms included generalized weakness, nausea, vomiting, lightheadedness, and oliguria. The patient was diagnosed with TSC at age three. She was found to have bilateral renal AMLs eight years prior. She was treated with everolimus for two years with insufficient shrinkage of the AMLs. She had a right nephrectomy four years prior due to the increasing size of the renal AML and development of a right-sided perinephric hematoma. A CT abdomen and pelvis showed the right renal AML measuring 16.1 cm x 12.0 cm x 19.6 cm with a right-sided perinephric hematoma (Figures 1, 2). Following nephrectomy, she was restarted on everolimus for the left-sided renal AML but stopped due to issues obtaining the medication. Over the course of her disease, she had multiple embolizations of the AML lesions. Her family history was significant for both her mother and brother, who had TSC.

**Figure 3:**
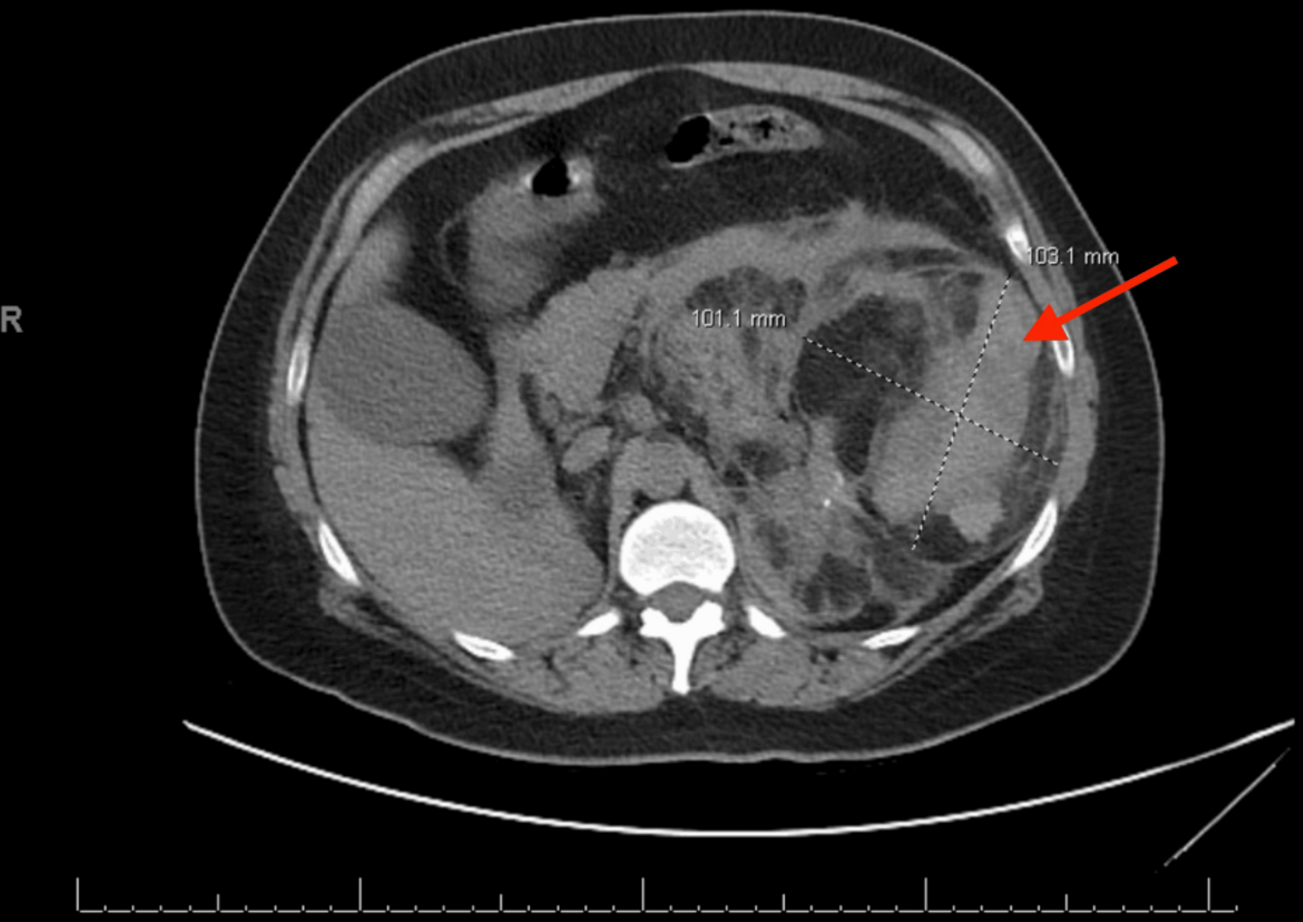
CT angiogram of the abdomen and pelvis: large left angiomyolipoma essentially replacing left renal parenchyma, measuring 12.8 x 12.8 x 21 cm with left perinephric hematoma in the axial plane

**Figure 4:**
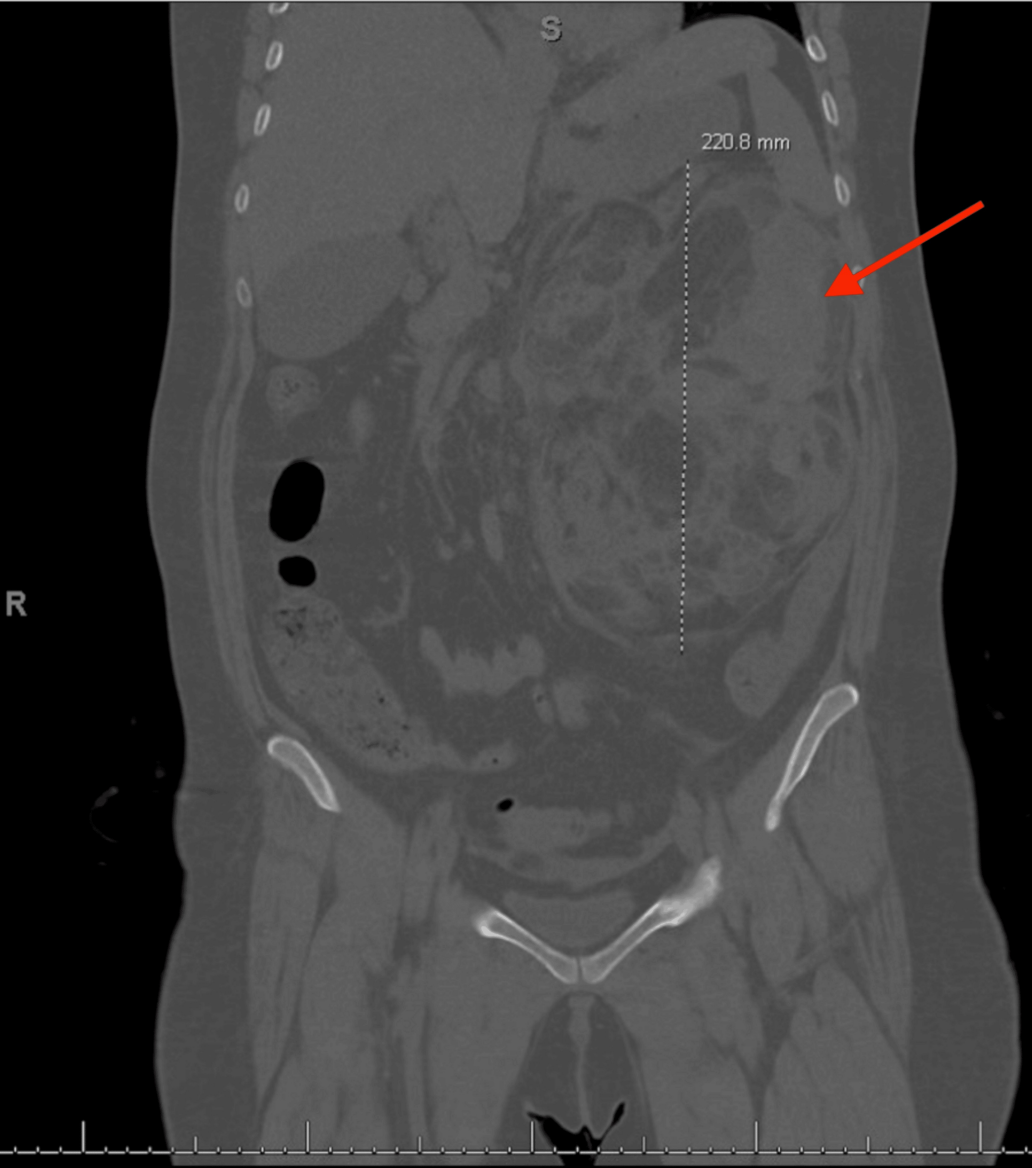
CT angiogram of the abdomen and pelvis: large left angiomyolipoma, measuring 12.8 x 12.8 x 21 cm with left perinephric hematoma in the coronal plane

Her vital signs on admission were within normal limits except for a heart rate of 124 beats per minute. Physical exam was significant for conjunctival pallor, diffuse abdominal tenderness to palpation, abdominal distension, and tachycardia. Her complete blood count showed a hemoglobin of 7.2 g/dL (reference range: Hb 12.2-16.4 g/dL). Her basic metabolic panel showed a creatinine of 3.17 (reference range: Cr of 0.6-1.1 mg/dL) (baseline GFR: 25). The patient's lab results on admission are presented in Table 1. A CT angiogram of the abdomen and pelvis showed a very large left AML measuring 12.8 x 12.8 x 21 cm with left perinephric hematoma without evidence of active extravasation (Figures 3, 4). The lesion was hypervascular and had essentially replaced all normal renal parenchyma on the left side, consistent with a high-risk hemorrhagic phenotype.

**Table 1:** Summary of the patient’s laboratory results on admission BUN: blood urea nitrogen; ALT: alanine transaminase; AST: aspartate transaminase

Laboratory test	Patient’s values	Reference range
Red blood cells	2.37 x 10^6^/μL	3.93-5.25 x 10^6^/μL
Hemoglobin	7.2 g/dL	12.2-16.4 g/dL
White blood cells	13.38 x 10^3^/μL	4.30-11.10 x 10^3^/μL
Platelets	397 x 10^3^/μL	166-358 x 10^3^/μL
Sodium	140 mmol/L	135-145 mmol/L
Potassium	4.2 mmol/L	3.5-5 mmol/L
Chloride	109 mmol/L	98-108 mmol/L
Bicarbonate	19 mmol/L	23-31 mmol/L
BUN	33 mg/dL	7-23 mg/dL
Creatinine	3.17 mg/dL	0.60-1.1 mg/dL
Total bilirubin	0.3 mg/dL	0.1-1.1 mg/dL
Albumin	3.9 g/dL	3.5-5.0 g/dL
Alkaline phosphatase	122 U/L	34-122 U/L
ALT	38 U/L	5-35 U/L
AST	38 U/L	13-40 U/L

The following morning, her hemoglobin acutely dropped to 6.3 g/dL (reference range: Hb of 12.2-16.4 g/dL), requiring transfusion of two units of packed red blood cells. Urology and interventional radiology were consulted, who recommended deferring embolization unless the patient became hemodynamically unstable or transfusion-dependent because of her young age and high risk of progression to end-stage renal disease and dialysis dependence. Her hemoglobin responded to transfusion and stabilized, while her renal function gradually returned to baseline. On hospital day four, the decision was made to discharge the patient home. She was referred to transplant surgery for eventual nephrectomy as an elective procedure based on her renal function trend. The decision to start dialysis would be determined outpatient by nephrology. She was also referred to medical oncology and urology.

## Discussion

Our case presents a delicate situation requiring close monitoring to prevent the initiation of hemodialysis in a young patient with a history of prior nephrectomy for the same indication. The incidence of renal AMLs is about 0.3-3% of all renal neoplasms, with 20% of cases associated with TSC. In the literature, very few “giant” renal AML cases have been documented, with the largest measuring about 39 cm reported in 2013 [9].

Medical therapy is preferred over surgical intervention to preserve renal function whenever possible [10]. Everolimus, an alternative to the interventional procedure, works directly by inhibiting mTOR1 to suppress protein translation and cell proliferation. The efficacy of everolimus in reducing the growth of AML has been reported in the EXIST-2 trial. In the EXIST-2 trial, 97% of patients on everolimus depicted stabilization or shrinkage of the AML, with 80.3% showing a reduction in size greater than 30% [11]. Based on these findings, the International Tuberous Sclerosis Complex Consensus Conference (ITSCCC) recommends the use of an mTOR inhibitor as first-line therapy for asymptomatic or growing AMLs larger than 3 cm [12]. NHS England guidelines recommended continued surveillance with MRI or CT after six months of everolimus treatment with continued monitoring for side effects and toxicity [11]. In our patient’s case, although everolimus was initiated, the response to everolimus was incomplete due to challenges in medication access, which likely facilitated tumor progression. Given her history of a prior nephrectomy, it is crucial for close surveillance and routine follow-up with oncology to monitor further progression of AML growth.

Patients with TSC can have a near-normal life expectancy, averaging around 70 years. The life expectancy can be altered by various disease processes in different organs and the extent of involvement. The leading cause of death is sudden unexpected death in epilepsy (SUDEP) that typically occurs at a younger age, with a median age of around 29 years [13]. The second most common cause of death is lymphangioleiomyomatosis (LAM), especially in women [13]. Renal complications from AMLs were historically a common cause of death in these patients. However, selective artery embolization and mTOR inhibitors have reduced complication rates and improved outcomes in TSC patients with renal AMLs, resulting in fewer deaths.

Renal tumors, especially renal AMLs, are the most common cause of perinephric hematomas. No clear guidelines currently exist regarding the management of perinephric hematomas. Once it has been diagnosed, treatment of a perinephric hematoma largely depends on the patient’s hemodynamic status, laboratory results, and the degree of underlying kidney injury. When AMLs become symptomatic or pose a hemorrhagic risk - particularly in tumors >4 cm or with aneurysms >5 mm - embolization is typically considered. However, in patients with advanced CKD or a solitary kidney, this carries a risk of renal infarction and progression to dialysis dependence. In cases of acute presentation, treatment often requires nephrectomy or embolization in hemodynamically unstable patients [14]. However, in our patient’s case, given their prior nephrectomy and age, a more conservative treatment was adopted to preserve the remaining kidney function. After hemodynamic stability was achieved with blood transfusions, the decision was made to adopt a conservative strategy rather than an invasive approach. Interestingly, during our literature review, we found one case of a patient with perinephric hematoma managed conservatively and underwent close monitoring on an outpatient basis [15]. The patient was followed for two years with close monitoring with regular blood pressure measurements, ultrasound imaging, and blood/urine studies, with eventual resolution of the hematoma. This conservative management plan avoids further renal compromise while ensuring the hematoma resolves over time [15]. The interdisciplinary approach to managing renal AMLs is primarily coordinated by oncology, nephrology, urology, and diagnostic/interventional radiology.

## Conclusions

This case highlights the complex management challenges in a young patient with TSC and giant renal AMLs complicated by perinephric hematoma in the setting of prior nephrectomy and CKD. In patients with TSC, preservation of renal function is vital, given the progressive nature of the disease and the risk of dialysis dependence. While everolimus remains the cornerstone of medical therapy for AMLs, its use may be limited due to incomplete response in certain individuals and barriers to medication access. The risk of dialysis dependence in a young patient with prior nephrectomy must also be carefully considered when evaluating an intervention such as embolization. This case underscores the importance of a multidisciplinary approach and balancing timely intervention with conservative management to optimize outcomes. Close outpatient follow-up with oncology, nephrology, and urology is essential to monitor disease progression, ensure medication access, and determine the appropriate timing of nephrectomy or initiation of dialysis, if necessary.
